# Ethanol Exacerbates the Alzheimer’s Disease Pathology in the 5xFAD Mouse Model

**DOI:** 10.3390/neuroglia5030020

**Published:** 2024-08-02

**Authors:** Hassan E. Mohammed, James C. Nelson, S. Alex Marshall

**Affiliations:** Department of Biological & Biomedical Sciences, North Carolina Central University, Durham, NC 27707, USA

**Keywords:** Alzheimer’s disease, alcohol use disorder, astrocytes, amyloid beta, microglial senescence

## Abstract

Alzheimer’s disease (AD) is the most common form of dementia with characteristic biological markers. Clinically, AD presents as declines in memory, reasoning, and decision making, but the loss of memory is particularly associated with hippocampal damage. Likewise, excessive ethanol consumption has been found to disrupt hippocampal function and integrity. To assess the potential shared consequences of AD pathology and ethanol, 5xFAD mice were administered 5 g/kg ethanol daily for 10 days. Immunohistochemical analysis revealed ethanol and AD converged to lead to microglial and astrocytic senescence as well as increased Aß-plaque formation in the hippocampus. Despite the exacerbation of these potential mechanisms of neurodegeneration, there were no additive effects of ethanol exposure and AD-related genotype on Fluoro-Jade C (FJC)+ cells or cognitive deficits in the novel object recognition task. Overall, these results are the first to characterize the effects of ethanol exposure on early adulthood in the 5xFAD mouse model. Together these findings support the idea that alcohol can influence AD pathology; however, the mechanisms involved in AD progression (e.g., glial activation and Aß-plaque) may be impacted prior to evidence of pathology (e.g., cognitive decline or neuronal loss).

## 1. Introduction

Alzheimer’s disease (AD) is the most common form of dementia marked by characteristic biomarkers, amyloid-ß (Aß) plaques and hyperphosphorylated tau protein aggregates in the brain [1]. Behaviorally, patients with AD also experience deficits in memory recall and spatial awareness [2,3]. As the global population ages, incidences of AD continue to increase, while cases and mortality rates due to other major diseases, such as heart disease and cancer are declining [4]. Increased life expectancy is a major contributing factor to the rise in dementia cases worldwide [5]. However, age is not the only life-factor that is thought to increase susceptibility in the development and progression of AD. Excessive ethanol consumption has been found to cause increases in the biological markers associated with AD [6]. For example, a preclinical study showed that ethanol exposure led to an increase in tau and Aß expression in an AD mouse model [7]. Currently over 6 million people in the United States suffer from AD, and cases are expected to rise to over 12 million by 2050 [8]. Considering over 40% of Americans over the age of 65 still consume alcohol [9], it is important to determine the potential influence of alcohol use on biomarkers associated with AD.

While the exact causes of AD progression are not fully clear, researchers have suggested some biomarkers that are characteristic of AD pathology [1,3,10]. One significant biological characteristic of AD is the aggregation of Aß plaques in the hippocampus and neocortex regions of the brain [11]. Aß plaques bind to receptor proteins and neuronal membranes, which is believed to cause inflammation and neurodegeneration [11]. Researchers believe that the early suppression of Aß clearance can lead to AD pathology downstream, but the exact mechanism is unknown [11]. The current work uses Aß accumulation in the 5xFAD mouse model of AD because it consists of five mutations related to the overexpression of Aß. Mice in the 5xFAD model express three mutations related to amyloid precursor protein (APP); a protein involved in Aß plaque accumulation [12]. 5xFAD mice also express two mutations in genes that express Presenilin-1 (PSEN1), a protein involved in the cleavage of APP into Aß [12,13]. By exposing 5xFAD mice to ethanol, the current work seeks to elucidate the influence of alcohol use disorders (AUDs) on Aß aggregation.

The accumulation of Aß is thought to facilitate neurodegeneration in AD, but glial senescence has emerged as an additional mechanism in AD progression. The hypothesis suggests that microglia and astrocytes in AD can promote neurodegeneration by becoming hyperactive and inflammatory while simultaneously having a subset of cells that are hypoactive and lose their ability to provide trophic support [14]. Moreover, the senescent state of microglia in aging and AD is thought to reduce their phagocytic capacity leading to an accumulation of damaged cells as well as the inability to remove protein aggregates like Aß [15,16]. In fact, studies have shown that amyloid beta can directly lead to these senescent phenotypes in both astrocytes [17] and microglia [18]. Likewise, some studies have indicated that ethanol leads to a similar dystrophic microglial state [19,20] and may disrupt the trophic support from glia in aging [21]. These dystrophic cells take on a distinct morphology and, in the case of microglia in AD, are associated with light-chain ferritin deposition [22–24]. In the current study, ferritin [25] and GFAP immunoreactivity [26] are used to examine microglial senescence and astrocytic responses, respectively.

The neurodegeneration in AD, whether related to Aß accumulation or glial senescence, ultimately results in cognitive and behavioral changes. The most prevalent of these changes is memory loss; patients affected by memory loss exhibit declines in spatial and temporal memory, language processing, and executive functioning [27]. Likewise, memory deficits from alcohol use occur during intoxication but also have long-term implications with repeated use [28,29]. In fact, alcohol is one of the leading causative factors in dementia, especially in early-onset dementia cases [30]. While there are several ways to assess learning and memory in rodent models, mice in the current study were assessed in the novel object recognition (NOR) task. The NOR task utilizes the understanding that mice are more likely to explore areas or objects to which they are unfamiliar. The NOR task examines whether a mouse will recognize a familiar object as familiar and spend less time exploring it as compared to the novel object. Previously, ethanol [31–33] and the genetic mutation in 5xFAD [34,35] have both been found to negatively impact the ability for mice to discriminate between novel and familiar objects, but the comorbid contributions of alcohol and Aß overexpression on learning and memory will be determined herein.

This study tests the hypothesis that excessive ethanol consumption exacerbates AD hippocampal neuropathology and behavior deficits. Others have looked at the progression of AD in relation to ethanol exposure in other AD models [7,31], but the current work adds to the field by determining alcohol’s effect in the 5xFAD mice. Moreover, this work uses immunohistochemistry to consider the hypoactive, senescent glial response that may also contribute to the enhanced neurodegeneration expected with alcohol exposure and AD pathology.

## 2. Materials and Methods

### 2.1. Animal Model

Male 5xFAD or wild-type (C57BL/6J) mice were used in the current study (Jackson Laboratory; Bar Harbor, ME, USA). A total of seventy-two mice were used for experimentation, but eight died prematurely. Mice had ad libitum access to water and Teklad Diet^®^ 7912X (Harlan Laboratories Inc.; Indianapolis, IN, USA) during experimentation. Mice were given nine days to acclimate to their environment before experimentation. All procedures used in this study were approved by the North Carolina Central University Animal Care and Use Committee (Approval number: SAM-11-22-2019) and followed the guidelines for the Care and Use of Laboratory Animals [36].

### 2.2. Ethanol Exposure Model

**Figure 1.**
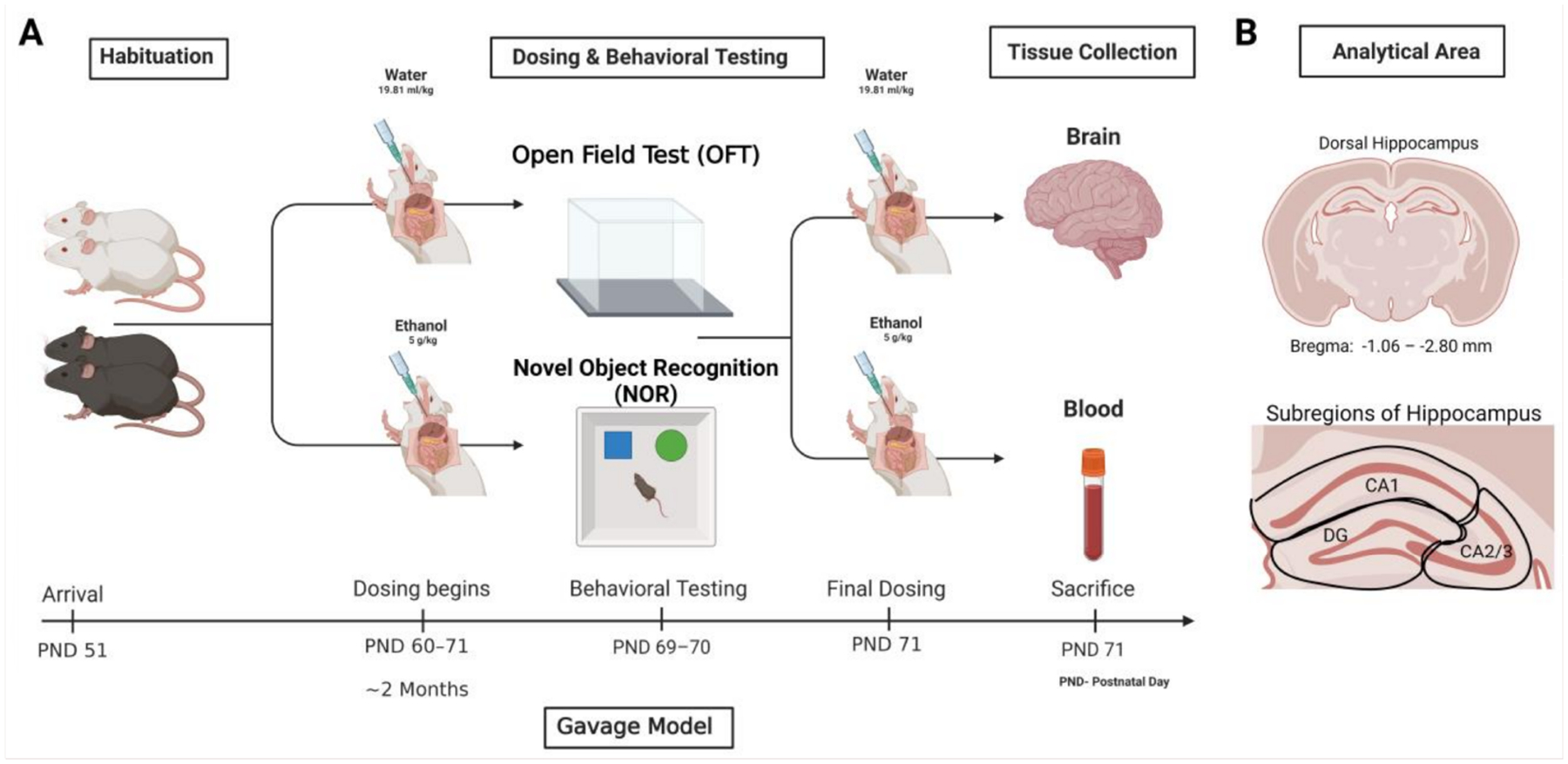
Schematic of gavage treatment, behavioral paradigm, and tissue collection procedures. (**A**). Images were taken of the dorsal hippocampus and the subregions of the hippocampus were assessed separately (**B**). This figure was made using BioRender.com.

Mice (PND 60) were dosed with ethanol 5 g/kg (25% *v*/*v*, Decon Labs, Inc.; King of Prussia, PA, USA) or isovolumetric water by intragastric gavage, for 10 consecutive days. The 10-day gavage model was used to mirror the effects of binge drinking at a rate that would induce alcohol-related brain damage (ARBD) in mice [37,38]. The daily gavage model was slightly modified so that mice were not dosed on behavioral test days to circumvent the acute effects of ethanol on behavior. However, mice were dosed 1 h before tissue collection to determine whether the genetic background influenced ethanol metabolism. See Figure 1 for additional details.

### 2.3. Immunohistochemistry

GFAP, l-ferritin, and Aß were assessed using immunohistochemistry with 3,3^′^-Diaminobenzidine (DAB) as a chromogen, similar to previous studies [7,21,39–41]. Immunohistochemistry assays were used because they allowed for the assessment of protein density in the cornu ammonis 1 (CA1), cornu ammonis 2/3 (CA 2/3), and dentate gyrus (DG) regions of the hippocampus. Brain tissue was sliced coronally at 40 μm-thick in a 1 in 4 series using a Compresstome VF-300 microtome (Precisionary Instruments INC., Greenville, NC, USA). Brain slices were stored in cryopreserve (Polyvinyl-pyrrolidone; Ethylene glycol; 0.2 M Phosphate Buffer) solution prior to immunohistochemical staining at −20 °C. To assess biological markers of AD and neurodegeneration, free-floating tissues of 2-month-old mice were washed with PBS, 3 times for 5 min, to remove excess cryopreserve. To block endogenous peroxidase activity, tissue was then incubated in 0.6% hydrogen peroxide (H_2_O_2_) in PBS followed by 3 × 10-min washes of PBS. Blocking of peroxidase activity reduces false positive DAB staining as peroxidase can bind DAB. To reduce the non-specific binding of primary antibodies, a 3% goat serum blocking solution in PBS and 1% Triton X-100 was used. Free-floating sections were then incubated in primary antibody rabbit anti-amyloid (1:1000) (Rockland Immunochemicals Inc.; Limerick, PA, USA), rabbit anti-ferritin light chain (1:1000) (Proteintech Group Inc.; Rosemont, IL, USA), or rabbit anti-GFAP (1:1000) (Invitrogen; Walthham, MA, USA) for 72 h at 4 °C. After incubation, tissue underwent 3 × 10-min washes of PBS to remove excess antibody that was not bound to the tissue. Tissue was then incubated in secondary biotinylated goat anti-rabbit antibody (1:2000) (Vector Laboratories; Newark, CA, USA) for 1 h and then washed 3 times for 10 min each before being incubated in Avidin-Biotin Complex (ABC) followed by 3 more PBS washes at 10 min each. ABC allows for the amplification of the biotinylated secondary. Lastly, free-floating tissues were treated with DAB for 1 min and 30 s until color change before being washed 3 times for 10 min in PBS. Samples were mounted in deionized water and coverslipped with Cytoseal^™^ (Thermo Fisher Scientific, Waltham, MA, USA) for imaging.

### 2.4. Immunoreactivity Quantification

For all imaging, a Zeiss Axio Observer 3 microscope and Axiocam 506 color camera (Zeiss Group, Dublin, CA, USA) with Zeiss Zen software (Blue 3.6) was used. All images were taken using a 20× objective. Image Pro 11 software (Media Cybernetics, Inc., Rockville, MD, USA) was used to determine cell counts and the general density of protein aggregates. GFAP, l-ferritin, and Aß were assessed using an immunoreactivity threshold (pixel intensity) as determined by the user, similar to our previous reports [40,41]. Hippocampal subregions, CA1, CA 2/3, and DG were traced to allow for separate assessments. Immunoreactivity was averaged across at least 5–6 images of sections and are expressed as percent area (+pixels/total area).

### 2.5. Fluorojade-C Staining

Brain tissue sections were mounted onto charged slides and dried overnight. Prior to Fluorojade-C (FJC) staining, tissues were pretreated. Pretreatment of tissue is necessary for probes to properly stain areas of interest. Slides were immersed into a basic alcohol solution mixture containing 5% NaOH in absolute ethanol for 5 min. Subsequently, slides were transferred to 70% ethanol, followed by water. Exposure to ethanol solutions dehydrates tissues by replacing the water molecules within. Subsequently, slides were transferred to a 0.06% potassium permanganate (KMnO_4_) solution followed by a series of de-ionized water (diH_2_O) washes. KMnO_4_ exposure reduces signal to noise, causing increased contrast of fluorescent staining for better visualization [42]. Slides were then submerged into 0.0001% FJC for 20 min in the dark, followed by a series of diH_2_O washes, placed on a drying rack, and then coverslipped using Vectashield mounting medium with 4^′^,6-diamidino-2-phenylindole (DAPI) (Vector Laboratories; Newark, CA, USA). Cell profile counts were assessed in the CA1, CA2/3 and DG hippocampal sub-regions. FJC+ cell counts are expressed as cell/section.

### 2.6. Open Field Testing

After 10 days of ethanol consumption by gavage, mice underwent behavioral testing in the open field test as previously reported [39]. The open field test has been used to measure anxiety-like behavior and locomotor activity in mice by assessing the total distance traveled and time spent in thigmotaxis [43]. Thigmotaxis in the open field test is defined as mice staying on the outer edges of the arena. Significant time spent in thigmotaxis is suggestive of anxious behavior in mice [43]. Open field testing was conducted to determine if there were any persisting effects of ethanol consumption and to assess whether there was any effect or interaction with Alzheimer’s disease pathology. For this test, mice were placed in a 40.64 × 40.64 × 30.48 cm plexiglass arena and allowed to explore for 10 min. Anxiety in mice was assessed based on the amount of time spent in thigmotaxis or the time spent on the outer edges of the open field. Mouse activity was recorded and later analyzed using Any-Maze 6.3 software (Stoelting Co.; Wood Dale, IL, USA). The total distance traveled (cm) was measured to determine any effects of treatment or genotype on locomotor activity. Time spent in thigmotaxis (s) was assessed to determine if there was an effect of treatment or genotype on anxiety. Anxiety-like behavior and locomotor activity were also evaluated because significant differences in locomotor activity and anxiety across experimental groups can have an impact in novel object recognition.

### 2.7. Novel Object Recognition

The novel object recognition (NOR) test was conducted to determine the memory recall of mice as previously reported [32]. Mice are believed to spend more time exploring novel objects that they are unfamiliar with compared to objects that they are familiar with [32,44]. The NOR test uses this understanding in determining a mouse’s recognition of a novel object when compared to a familiar object based on exploration time to understand memory recall. As previously stated, ethanol consumption and Alzheimer’s disease pathology have been found to impact memory function independently in mice [31,45,46]. In the first stage of NOR, smooth wooden spheres ¾′′ in diameter (Woodworks Ltd.; Fort Worth, TX, USA; Item#: BE1070) were placed into each home cage of mice to allow for habituation toward the object known as a familiar object for 24 h prior to testing. Importantly, the familiarization period occurred the day after the 10th gavage so that animals were no longer intoxicated, as previous studies have shown that intoxication disrupts memory encoding and consolidation [47,48]. On testing day, mice were individually assessed on their recognition of the familiar object against a new novel object of a different texture, a sphere with honeycomb texture also ¾′′ in diameter (Woodworks Ltd.; Item#: BE6070). Mouse activity was recorded and later assessed using Any-Maze 6.3 software (Stoelting Co.). To score item preference, the discrimination index was evaluated. Analysis of the discrimination index accounts for differences in the total exploration time between individual mice. The discrimination index considers the total time spent exploring both objects as well as each object individually [32].

### 2.8. Intracardiac Perfusions

Similar to previous studies, intracardiac perfusion was conducted to euthanize mice in order to maintain the integrity of brain tissues for immunohistochemical analysis [39,40]. Perfusions were conducted 1 day after behavioral testing. First, mice were injected with ~0.15 mL of xylazine (10 mg/kg) and ketamine (100 mg/kg) in saline solution intraperitoneally to induce anesthesia. A phosphate buffered saline (PBS, pH = 7.4) solution was then flushed through the body to wash out the blood in the vasculature. After 3 min of PBS perfusion, 4% paraformaldehyde was flushed throughout the body to fix tissue. Brains were retrieved and stored in 4% paraformaldehyde for 24 h followed by PBS for storage in 4 °C.

### 2.9. Blood Ethanol Concentration Determination

After the final dose of ethanol, mice were sacrificed using intracardiac perfusion, and trunk blood was collected. Plasma was isolated by centrifugation at 1500× *g* for 5 min and immediately stored at −20 °C. BECs were quantified using the EnzyChrom^™^ Ethanol Assay Kit (BioAssay Systems, Hayward, CA, USA), a colorimetric assay, run in duplicate. Absorbances were read at 565 nm using the Synergy HTX Microplate Reader (BioTek Instruments, Inc., Winooski, VT, USA) and averaged within each individual mouse.

### 2.10. Statistical Analysis

All data were graphed and analyzed using GraphPad Prism 9.0 (San Diego, CA, USA). BECs were compared using a *t*-test to determine if genotype impacted ethanol pharmacokinetics. All other data were analyzed using two-way analyses of variance (ANOVAs; treatment × genotype). Effects were considered significant if *p* < 0.05. Tukey’s post hoc analyses were used if an interaction or a main effect of both alcohol and genotype were observed. The decision to use post hoc analyses when both main effects were seen was based on a priori hypotheses that ethanol and genotype would independently influence many of our measures [49]. All data presented in the figures are the mean ± SEM.

## 3. Results

### 3.1. Genotype Did Not Influence Blood Ethanol Concentrations

All animals received the same dose of ethanol, and no effect of genotype was determined by a *t*-test [t(9) = 0.56, *p* = 0.40] on the blood ethanol concentrations between the wild-type (μ = 129.0 ± 8.1 mg/dL) and 5xFAD (μ = 160.2.0 ± 13.22 mg/dL) animals.

### 3.2. 5xFAD Mice Were Resistant to Ethanol’s Effects on GFAP Immunoreactivity

**Figure 2.**
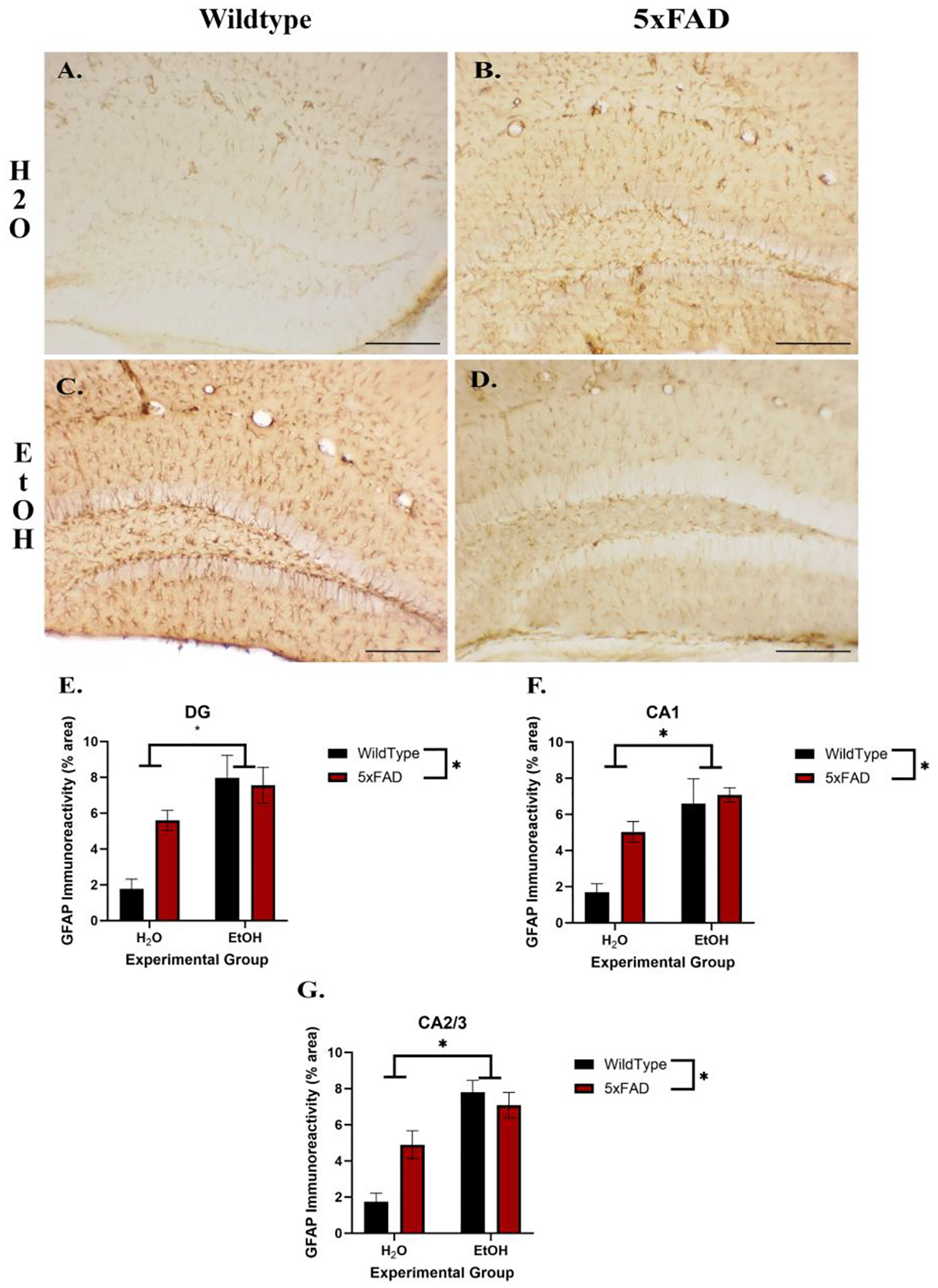
Photomicrographs suggests glial fibrillary acidic protein (GFAP) immunoreactivity (IR) is increased in the dentate gyrus (DG) by 5xFAD genotype (**B**) and ethanol (**C**) compared to the wildtype (**A**). There did not appear to be an additive effect in 5xFAD mice exposed to ethanol (**D**). Two-way ANOVA on GFAP immunoreactivity (IR) revealed a main effect of ethanol as well as genotype exposure on increased GFAP+ pixels in the DG (**E**), CA1 (**F**), and CA2/3 (**G**) regions of the hippocampus. Interestingly, there was no additive effect of GFAP IR when ethanol and Alzheimer’s genotypic susceptibility were combined. Scale bar = 10 μm; * *p* < 0.05 main effect.

The effects of ethanol exposure and AD pathology on GFAP immunoreactivity were assessed by measuring densitometry as a characteristic of astrogliosis (see Figure 2). In the CA1, a two-way ANOVA revealed a significant effect of genotype [F (1, 15) = 6.55, *p* = 0.02] as well as a main effect of ethanol [F (1, 15) = 21.85, *p* < 0.001] but no interaction [F (1, 15) = 3.69, *p* = 0.07]. For the CA2/3 and DG, two-way ANOVAs also revealed a significant effect of ethanol exposure (CA2/3: ([F (1, 15) = 32.50, *p* < 0.001], DG: ([F (1, 15) = 22.23, *p* < 0.001]) and an interaction (CA2/3: [F (1, 15) = 7.13, *p* = 0.02], DG: ([F (1, 15) = 5.94, *p* = 0.03]) but no main effect of genotype (CA2/3: [F (1, 15) = 2.87, *p* = 0.11], DG: ([F (1, 15) = 3.87, *p* = 0.07]). Post hoc analyses for all of the subregions suggested that the water–5xFAD mice were similar to the ethanol-treated animals regardless of genotype, indicating that the astrocytes of the 5xFAD mice may not be as responsive to ethanol as the wild-type animals.

### 3.3. Increased L-Ferritin Immunoreactivity in Response to Ethanol Exposure and Genotype

**Figure 3.**
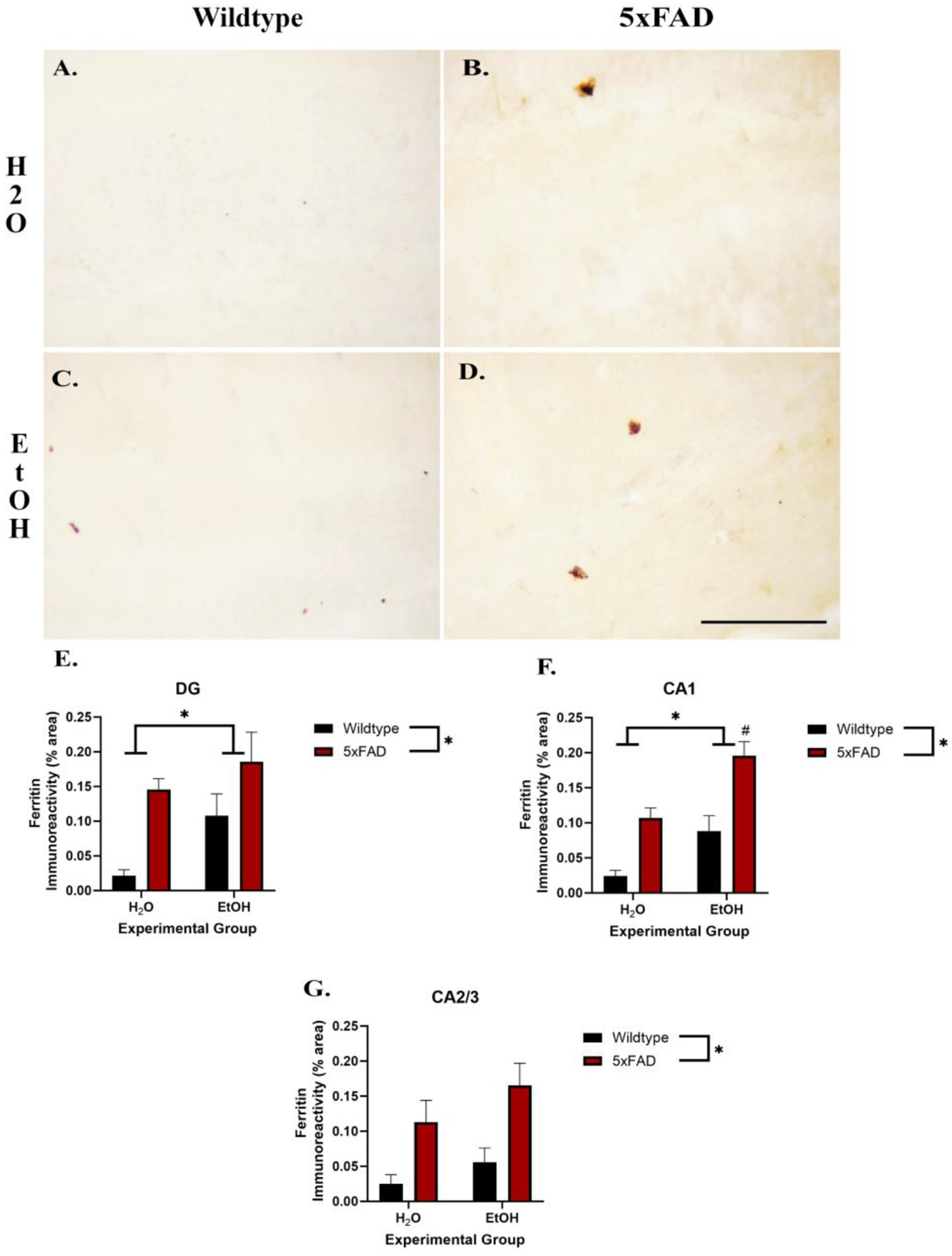
Photomicrographs of l-ferritin in the CA1 indicate that the 5xFAD genotype (**B**) and ethanol (**C**) lead to an increase in ferritin IR and appear to be additive when combined (**D**) compared with the control wildtype animals (**A**). Two-way ANOVA revealed a main effect of ethanol as well as genotype exposure on increased IR+ pixels in the DG (**E**) and CA1 (**F**) but only genotype in the CA2/3 (**G**) regions of the hippocampus. Scale bar = 10 μm; * *p* < 0.05 main effect. # < 0.05 post hoc comparison compared to 5xFAD and EtOH alone.

To measure the effects of ethanol exposure and genotype on l-ferritin secretion, l-ferritin IR was assessed (see Figure 3). Two-way ANOVAs revealed a main effect of genotype in all three subregions, indicating that the 5xFAD mice had a greater ferritin density than controls (CA1: [F (1, 15) = 30.11, *p* < 0.0001], CA2/3: [F (1, 15) = 11.95, *p* = 0.003], DG: [F (1, 15) = 11.77, *p* = 0.004]). However, only the CA1 [F (1, 15) = 19.41, *p* < 0.001] and DG [F (1, 15) = 4.55, *p* = 0.049] had a main effect of ethanol exposure, but no regions had an interaction (CA1: [F (1, 15) = 0.50, *p* = 0.49], CA2/3: [F (1, 15) = 0.15, *p* = 0.71] DG: [F (1, 15) = 0.69, *p* = 0.42]). Only in the CA1 did post hoc analyses indicate any additive effects of ethanol and genotype. More specifically, there was a significant difference between the water and ethanol treatment among the 5xFAD groups as well as an effect of genotype on the ethanol groups.

### 3.4. Ethanol Led to Increased Amyloid-Beta Deposition in the DG of 5xFAD Mice

**Figure 4.**
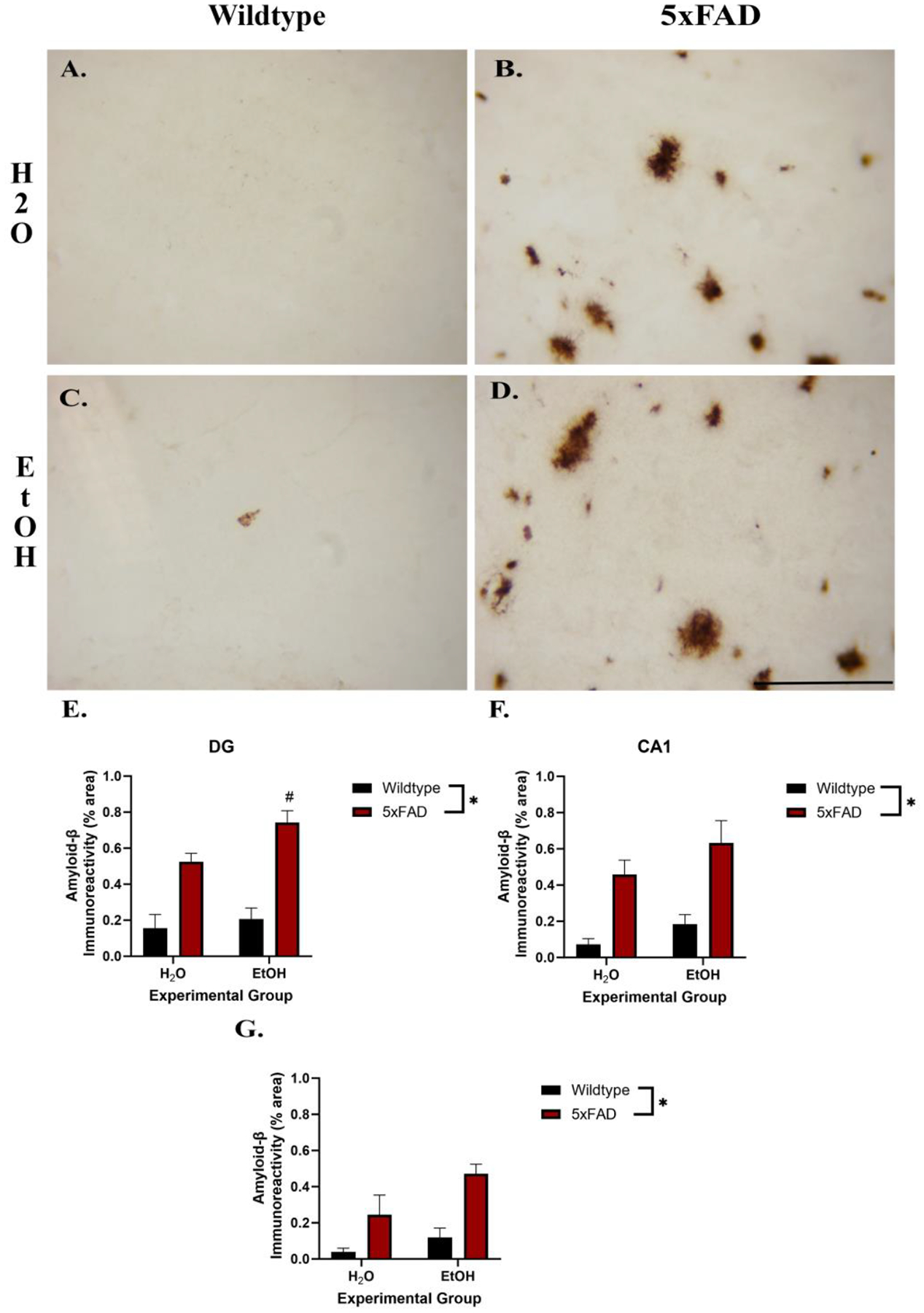
Photomicrographs of amyloid beta in the DG indicate that the 5xFAD genotype (**B**) led to an increase in amyloid beta deposition. Interestingly, alcohol increased this effect in the 5xFAD mice (**D**). There was very little to no amyloid beta in the wildtype animals exposed to water (**A**) or ethanol (**C**). Two-way ANOVA revealed a main effect of genotype exposure on increased amyloid beta+ pixels in the DG (**E**), CA1 (**F**), and CA2/3 (**G**). However, the DG post hoc tests suggest that ethanol had an exacerbating effect on amyloid in the DG (**E**). Scale bar = 10 μm; * *p* < 0.05 main effect. # < 0.05 post hoc comparison compared to 5xFAD alone.

Aß density was measured to examine the effects of ethanol consumption and genotype on Alzheimer’s disease pathology (see Figure 4). Two-way ANOVA revealed a significant effect of genotype on Aß throughout the hippocampus (CA1: [F (1,15) =22.17, *p* < 0.001], CA2/3: [F (1,15) = 12.05, *p* = 0.003], DG: [F (1, 15) = 54.01, *p* < 0.0001]), but no interactions were revealed (CA1: [F (1,15) = 0.13, *p* = 0.73], CA2/3: [F (1,15) =0.82, *p* = 0.38], DG: [F (1, 15) = 1.79, *p* = 0.21]). Only in the DG [F (1, 15) = 4.76, *p* = 0.045], but not the other hippocampal subregions (CA1: [F (1,15) =2.63, *p* = 0.12], CA2/3: [F (1,15) = 3.64, *p* = 0.08]), was there a main effect of ethanol. Tukey’s post hoc tests of the DG ANOVA indicated that there was an additive effect of ethanol on amylid density in addition to the expected increase associated with the 5xFAD genotype.

### 3.5. Ethanol and 5xFAD Genotype Caused an Increase in Fluorojade-C+ Cells in the Hippocampus

**Figure 5.**
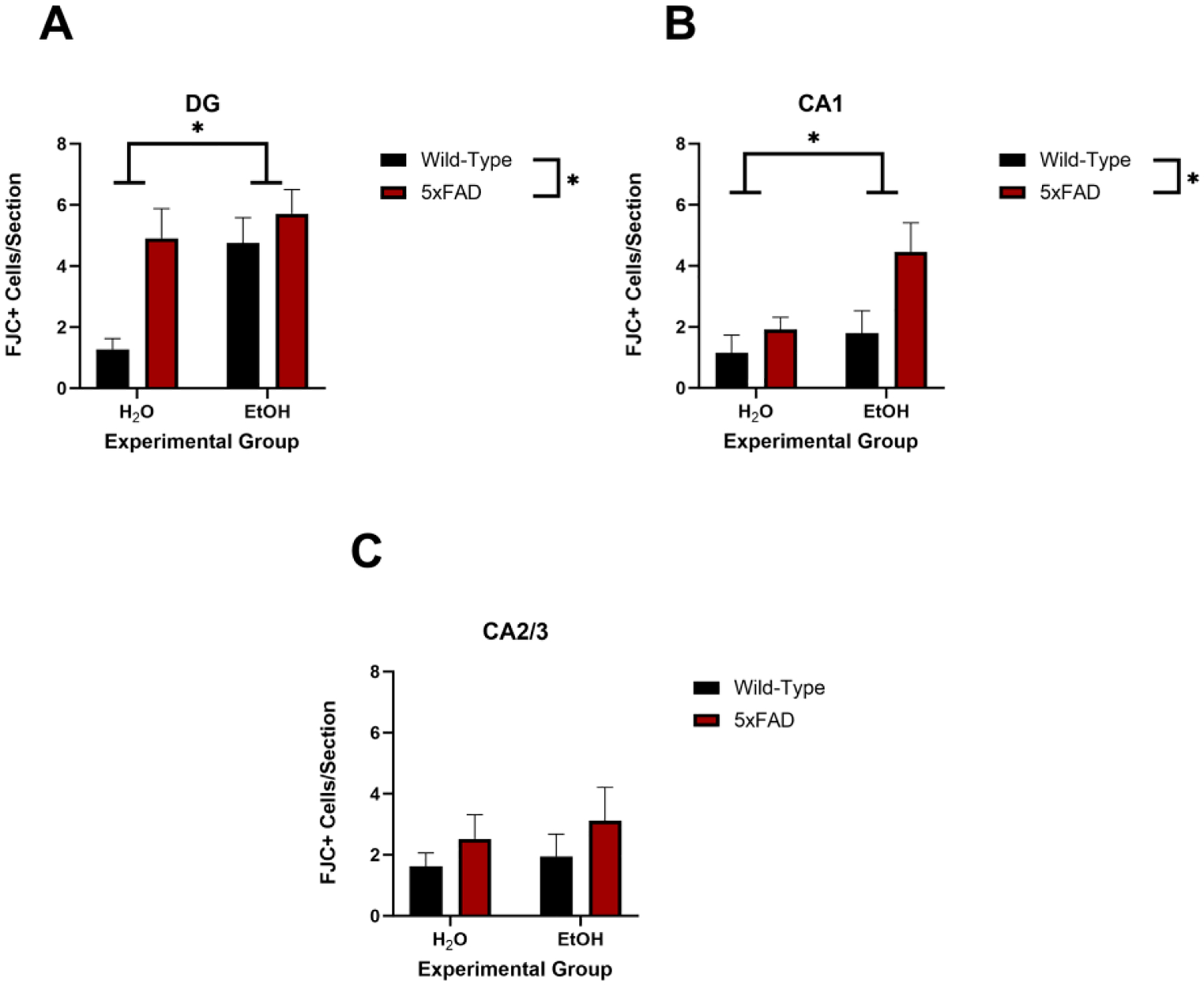
Fluorojade C+ cells were increase in the DG (**A**) and CA1 (**B**) by both ethanol exposure and the 5xFAD genotype. No effect was observed in the CA2/3 region (**C**). * *p* < 0.05 main effect.

FJC+ cell numbers were assessed to determine the effects of ethanol exposure and AD-related genotype on neurodegeneration (see Figure 5). In the CA1 and DG subregions, two-way ANOVAs revealed significant main effects of genotype (CA1: [F (1, 15) = 6.02, *p* = 0.026]; DG: [F (1, 15) = 7.70, *p* = 0.014]) and ethanol (CA1: [F (1, 15) = 5.21, *p* = 0.038]; DG: [F (1, 15) = 6.73, *p* = 0.020]) on increased FJC+ cells; however, no interaction was determined between these main effects in either the CA1 [F (1, 15) = 1.86, *p* = 0.19] or DG [F (1, 15) =2.63, *p* = 0.13]) subregions. Additional post hoc analyses did not determine any additive effects of the combination of ethanol and 5xFAD genotypes. In the CA2/3, neither ethanol [F (1, 15) = 0.29, *p* = 0.59] nor genotype [F (1, 15) = 1.47, *p* = 0.24] had a main effect on the number of FJC+ cells.

### 3.6. No Effects of Genotype or Ethanol on Anxiety-like Behavior

**Figure 6.**
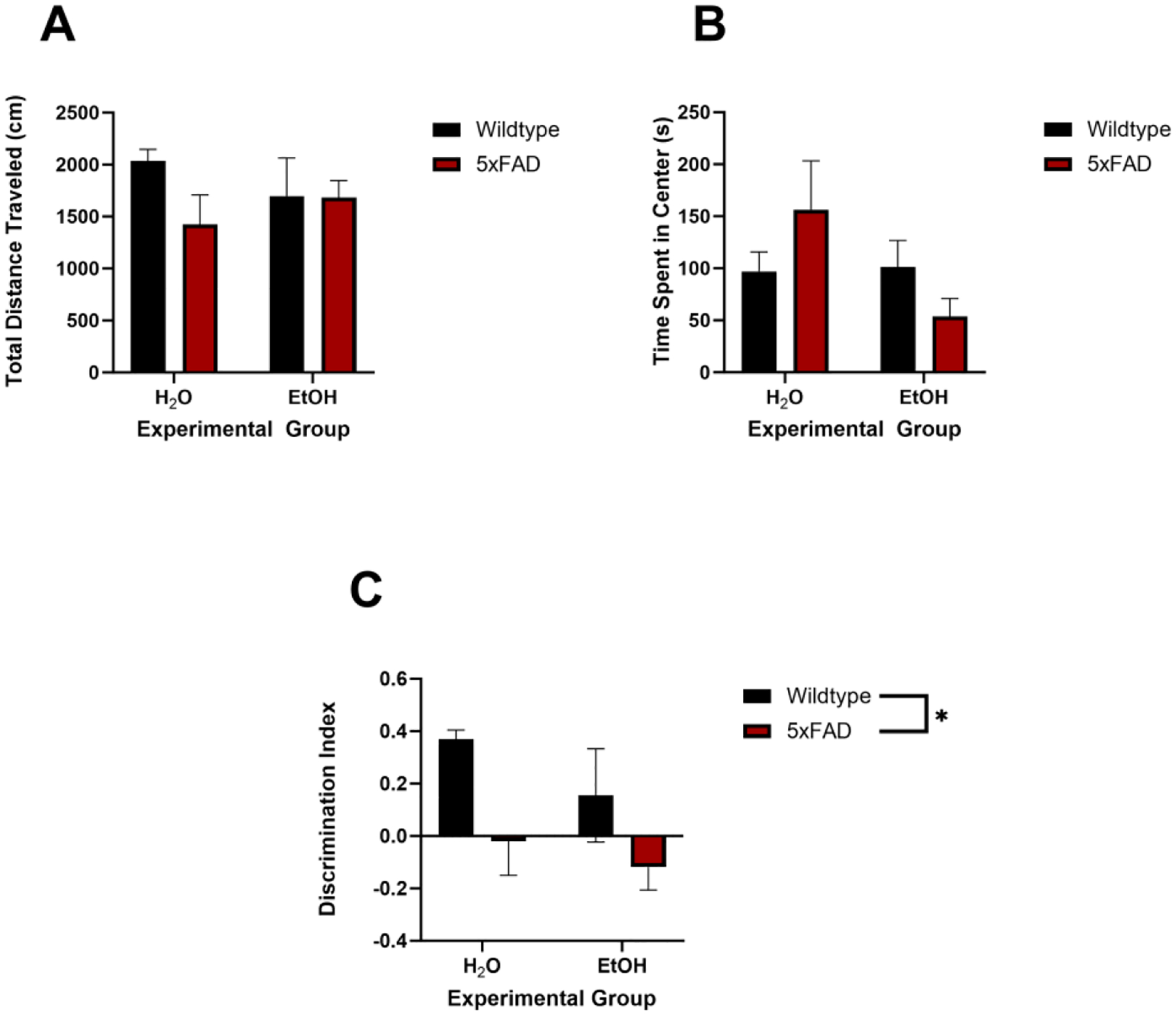
No effect of ethanol or genotype was observed on locomotor activity (**A**) or anxiety-like behavior (**B**) in the open field test. As expected, the 5xFAD mice did show cognitive decline as measured by the discrimination index in the novel object recognition task (**C**). * *p* < 0.05 main effect.

To measure the combined effects of ethanol exposure and AD pathology on anxiety and locomotor activity, performance in the open field test was assessed (see Figure 6). Total distance was used as a correlate of locomotor activity. Two-way ANOVA revealed no main effect of genotype [F (1, 15) = 1.44, *p* = 0.25] or ethanol exposure [F (1, 15) = 0.025, *p* = 0.88] nor any interaction [F (1, 15) = 1.33, *p* = 0.26] on the total distance traveled in the OFT arena. Likewise, two-way ANOVA revealed no main effect of genotype [F (1, 15) = 0.030, *p* = 0.87] or ethanol exposure [F (1, 15) = 2.00, *p* = 0.18] nor any interaction [F (1, 15) = 2.37, *p* = 0.14] on the time spent in the center of the OFT arena.

### 3.7. Genotype but Not Ethanol Influence Novel Object Recognition

To determine the effects of ethanol exposure and genotype on memory recall, mouse performance in the NOR test was assessed using the discrimination index (see Figure 6C). The discrimination index considers the total time spent exploring both objects as well as each object individually [32]. Two-way ANOVA revealed only a main effect of genotype [F (1, 15) =1.44, *p* = 0.25] but no main effect of ethanol exposure [F (1, 15) =1.62, *p* = 0.22] nor any interaction [F (1, 15) =0.22, *p* = 0.66] on the discrimination index.

## 4. Discussion

Excessive ethanol consumption has several consequences including dysregulated memory pathways, increased neuroimmune response, and neurodegeneration. Understanding the convergence of excessive ethanol consumption with pathological aging is important as aging populations continue to drink excessively [49,50]. The current study seeks to examine the potential for ethanol consumption to exacerbate the progression of AD pathology using a mouse model. The major findings from this manuscript are as follows: (1) ethanol and genotype led to an increase in astrocytic activation as shown by GFAP+ immunoreactivity, but 5xFAD mice seemed to reach a ceiling response and were no longer responsive to ethanol; (2) ethanol increased microglial dystrophy and Aß density due to genotype in the hippocampus as suggested by l-ferritin deposition; (3) increased Aß density as a result of 5xFAD; and (4) AD-pathology and ethanol lead to an increase in neurodegenerative FJC+ stained cells. However, despite these biological effects of ethanol on AD pathology, (5) there was no effect of ethanol exposure on deficits in memory recognition in 5xFAD mice.

Chronic astrogliosis is a potential consequence of excessive ethanol consumption. Previous studies have shown an effect of excessive ethanol consumption resulting in increased astrocytic activity [51,52]. Presently, increased GFAP immunoreactivity was shown in the CA1, CA2/3, and DG regions of the hippocampus, signifying increased astrocyte activity due to ethanol exposure. These results revealed increased gliosis as a response to excessive ethanol exposure that is congruent with past findings that ethanol consumption increases astrocytic activity in the hippocampus [53–55]. Dysregulation in astrocyte activity is also implicated in the development of neurodegenerative disorders, such as AD, as previously mentioned. Research shows the involvement of astrocytes in the clearance of AD-associated Aß plaques [56]. A further increase in GFAP expression with the introduction of ethanol in the presence of the 5xFAD genotype was expected, due to the proposed shared mechanisms of ethanol consumption and AD-related pathologies and the involvement of astrocytes in the neuroimmune response. However, the astrocytic response seemed to reach a ceiling effect when ethanol was added similar to our findings in aged animals [21]. A hyporeactive or senescent astrocytic response has also been observed in AD pathology and can lead to neurodegeneration [17,57]. Astrocytic senescence could impact glutamate regulation (leading to glutamate excitotoxicity), blood–brain barrier integrity, and a discoordination of neuroimmune responses [14,57]. The current results suggest that astrocytes may no longer be responsive to ethanol in the presence of AD pathology. Future studies are necessary to determine if the lack of astrocytic responses to ethanol have direct functional implications that would influence AD.

Astrocytic hypoactivation is not the only glial pathway that can lead to neurodegeneration in the brain. Dysregulation of l-ferritin storage by microglia has also been implicated in the hypo-activation of microglia and subsequent neurodegeneration. Increased l-ferritin is a marker of dystrophic microglia and increased oxidative stress [23–25]. Microglia are involved in the maintenance of ferritin and iron storage in response to oxidative stress [24,25,58,59]. Microglia store oxidized iron (FE^3+^) in l-ferritin molecules. High expression levels of l-ferritin in tissue are indicative of oxidation and a marker for FE^3+^ storage in microglia. In AD patients, increased expression of l-ferritin in microglia was found to be associated with dystrophic microglia [24]. Immunohistochemical analysis revealed increased expression of l-ferritin due to ethanol consumption in the CA1 and DG regions of the hippocampus, suggestive of an environment that would have oxidative stress and microglial dystrophy. While little research has been conducted on the roles of ethanol exposure on increased iron deposition specific to the hippocampus, it has been repeatedly suggested that Alzheimer’s is associated with a dysregulation of iron in microglia that leads to senescence [16,22,60–63]. The current work showed that at least in the CA1 the combination of AD predisposition and excessive alcohol consumption led to increased ferritin deposition. One proposed mechanism is that the presence of Aß and related proteins (APP and PSEN-1) causes a disruption in iron storage and homeostasis. The binding of iron by amyloid-beta and related proteins has been implicated in the increased oxidation and reduction of both FE^2+^ and FE^3+^, respectively, causing a disruption in oxidative homeostasis within the brain, but the exact mechanisms are unknown [64]. The potential additive effect of alcohol on ferritin deposition is particularly problematic in light of our preclinical studies which suggest that alcohol can independently lead to dystrophic microglia in the hippocampus [19]. The implications of these findings are further supported by clinical studies of other brain regions that have shown an effect of increased iron deposition in the brain due to ethanol consumption [65]. Thus, the findings presented suggest that ethanol consumption may exacerbate AD pathology by increasing iron deposition and the subsequent hypoactivation of microglia. These alcohol-induced increases in l-ferritin loaded microglia have the potential to lead to more oxidative stress and decreased neurotrophic support from microglia.

Aß has been repeatedly indicated as a biomarker of AD. Previous results suggest the accumulation of Aß in 5xFAD mice starts as early as 2 months, beginning in the subiculum and spreading to other regions, such as the hippocampus, as the mice age [12]. The present findings also confirm these findings that the overexpression of APP and presinillin-1 lead to increased Aß production early in life. Due to the proposed interactions between ethanol consumption and AD pathology, there was an expectation for significantly increased Aß deposits in 5xFAD mice that were exposed to ethanol. The results revealed increased responsiveness of Aß to ethanol exposure in the DG region of the hippocampus. The current results did not show an exacerbation of AB plaque formation due to ethanol consumption in the other subregions, but the DG has previously been shown to be more susceptible to ethanol compared to the other subregions of the hippocampus [41,66]. The accumulation of Aß in response to ethanol is the most direct evidence presented herein that indicates that ethanol may exacerbate AD pathology. These findings align with others studies examining the amyloid increase in AD transgenic models including 3xTg-AD [31,67], APPswe/PS1dE9 [68], and APP/PS1 mice [69]. The current work focused on Aß as the 5xFAD genetic manipulations are mainly associated with Aß deposition [12], but future work should also examine phosphorylated-tau after exposure to alcohol within the 5xFAD model, as it has also been shown to be upregulated with age in these transgenic animals [70,71]. However, this is the first set of experiments to examine ethanol’s influence on AD pathology in 5xFAD mice.

To further understand the neurodegenerative potential of Aß and ethanol consumption, FJC staining was utilized. Previously, researchers found increased fluoro-jade B+ cells, a precursor to FJC, in the dentate gyrus of 8-week-old mice after 10-day ethanol exposure [72]. In the current study, the results presented show increased FJC+ cells in the CA1 and DG regions of the hippocampus due to genotype. These results are indicative of an influence of 5xFAD-related pathologies on neurodegeneration, signs of which can be detected as of 2 months of age in the 5xFAD mouse model. Whilst AD pathology has been attributed to increased neurodegeneration, significant increases in FJC staining have not been previously found in this model as early as 2 months of age. It was expected that an interaction between the 5xFAD genotype and ethanol exposure would yield increased neurodegenerative FJC staining due to the proposed shared mechanisms of neurodegeneration between ethanol exposure and AD pathology [45]. However, despite the convergence of ethanol and AD pathology on glial dysfunction and amyloid beta, there were no combined effects of ethanol exposure and Aß overexpression. The single snapshot in this experiment of pathology at 2 months may not be sufficient to fully understand the implications of AD and alcohol use, but the combination of the neurodegenerative mechanisms still suggests that excessive alcohol consumption may be a critical factor for increased neurodegeneration within the hippocampus with aging and/or AD-related pathology.

Dysregulation of the hippocampus, such as is associated with both AUDs and AD, leads to disruptions in associated memory pathways. Ethanol exposure in mice has been associated with decreased performance in the novel object recognition (NOR) task. Previous research suggests a diminished performance in the NOR task due to adolescent ethanol exposure, as indicated by significantly lower discrimination index scores [33]. However, other studies show no significance of ethanol exposure on NOR performance [31,73]. Herein, there was no effect of ethanol on NOR, but the current results showed an influence of the 5xFAD genotype on discrimination index scores in the novel object recognition task. Our findings in NOR were not due to locomotor activity or anxiety-like behavior as shown in our OFT results. It is important to consider that the discrimination index for the 5xFAD mice suggests there was no preference for either object, and their interactions were closer to chance [74]. As such, not discovering an ethanol effect could simply be due to a floor effect. More sensitive assays of memory, such as an NOR task with multiple familiar objects, should be performed in the future; additionally, a more comprehensive battery of behavioral assessments that measure cognitive flexibility should be performed to truly assess whether ethanol may accelerate AD-related cognitive decline [44,75,76].

Collectively, the potential for ethanol consumption to induce or exacerbate the effects of AD pathology has been studied using various mouse models; however, this study examines the potential for ethanol to exacerbate Aß-related AD pathology using the 5xFAD mouse model. The results presented reinforce past findings that ethanol exposure and Aß expression led to increased hypo-reactive glial cells, as shown by the lack of increase in GFAP immunoreactivity in the ethanol-treated 5xFAD mice as well as the increase in ferritin deposition. However, there are several limitations to the current studies including the fact that these studies were all completed in male mice. Previous research has shown that both ethanol- and AD-associated neurodegenerative mechanisms can be influenced by sex [41,77–79]. Currently, studies examining the influence of sex on alcohol’s role in AD pathologies remain inconclusive, with some showing more robust changes in females [80–83], others indicating no direct influence [84], and lastly, some indicating worse outcomes in males [85]. Another important limitation of the current work is that it only uses one marker for microglia, astrocytes, neurodegeneration, and AD pathology, but future studies should thoroughly assess the microglial [86,87] and astrocytic [88,89] phenotypes to understand the combined effects of alcohol and AD on the pro- vs. anti-inflammatory state within the continuum of microglial activation. Moreover, FJC does not allow for a differentiation between the types of neurodegenerative processes, but future work could examine caspase 3, pRIP3, and/or TUNEL. Glial responses to injury and the associated mechanisms are key in understanding potential methods for combating neurodegenerative conditions, such as AD and AUDs. It is possible that this glial dysfunction led to an increase in Aß expression after alcohol exposure despite the lack of changes in neurodegeneration or behavioral outcomes. Future studies are necessary to understand the long-term potential synergism of AD pathology and ethanol exposure including looking at a host of additional variables including sex [90], the influence of stress [91], and drinking patterns [92] within the scope of the 5xFAD mouse model.

## Data Availability

The data supporting the conclusions of this article will be made available by the authors upon reasonable request.
